# Hepatitis B Virus Infection among Health Care Workers in Indonesia

**DOI:** 10.5005/jp-journals-10018-1269

**Published:** 2018-05-01

**Authors:** David H Muljono, Teguh Wijayadi, Rizalinda Sjahril

**Affiliations:** 1 Eijkman Institute for Molecular Biology, Jakarta, Indonesia; Universitas Hasanuddin, Makassar, Indonesia; and Sydney Medical School University of Sydney, New South Wales, Australia; 2 Universitas Hasanuddin, Makassar, Indonesia; Tarumanegara University, Jakarta Indonesia; 3 Universitas Hasanuddin, Makassar, Indonesia

**Keywords:** Health care workers, Hepatitis B, Hepatitis B virus, Occupational hazard, Occupational risk.

## Abstract

Hepatitis B virus (HBV) infection is a global health problem with an estimated 257 million chronically infected people. Indonesia is a moderately hepatitis B-endemic country with 7.1% prevalence of hepatitis surface antigen (HBsAg). This infection is considered as an important occupational hazard among health care workers (HCWs), who may become further transmitters of this infection. The extent of hepatitis B among HCWs and specific control strategy are not available in Indonesia. A study was done on 644 HCWs, who were categorized into administration, nonintervention, and intervention groups. The prevalence of HBsAg, antibody to HBV core antigen (anti-HBc), and antibody to HBsAg (anti-HBs) was 4.7, 18.5, and 36.7% respectively, while 57.3% were negative for all seromarkers, indicating susceptibility to this infection. Increasing trend with age was observed in the exposure to infection (anti-HBc) (p <0.001) and the marker of resolved infection (HBsAg-, anti-HBc+, anti-HBs+) (p = 0.004), suggesting accumulated exposure to HBV infection by increasing age. Rising trend of exposure rate was also observed across the administration, nonintervention, and intervention groups (p < 0.001). By length of service period, significant escalation of exposure (p = 0.010) and resolved infection (p < 0.001) were also observed, suggesting increasing occupational risk to HBV infection. There is an urgent need to safeguard the HCWs with hepatitis B vaccination and provide continuing education at various health care setups. The establishment of a national policy and a roadmap for effective and efficient intervention is required for the prevention, diagnosis, postexposure management, and treatment of HBV infection in this special population.

**How to cite this article:** Muljono DH, Wijayadi T, Sjahril R. Hepatitis B Virus Infection among Health Care Workers in Indonesia. Euroasian J Hepato-Gastroenterol 2018;8(1):88-92.

## INTRODUCTION

The global prevalence of HBV infection in the general population is estimated to be at 3.5% with about 257 million people living with chronic HBV infection. This infection accounts for 887,000 annual deaths due to its complications, including cirrhosis and hepatocellular carcinoma.^1^ Indonesia with a total population of more than 250 million people has 7.1% prevalence of HBsAg, and, therefore, is classified as a moderately hepatitis B-endemic country.^2^

Several groups have been assigned as special populations who have particular risks for acquiring HBV infection. Among those are HCWs who are at high risk of occupational hazard, and may further transmit this virus to patients and their families.^3,4^ It has been shown that HCWs have an up to four-fold incidence of this infection in the general population. The main risk factor to acquire HBV infection for HCWs is direct contact with infectious material, especially HBV-infected blood or body fluid.^5^ Some studies also have reported that awareness of HBV and proper precautions against blood-borne infections are lacking in these workers.^6^

Recommendations have been formulated in parallel with public health and clinical efforts to combat this disease globally. Nevertheless, no formal survey and measures of occupational exposures have been formulated for HCWs in many hepatitis B-endemic countries including Indonesia.^3,7-12^ This review examines the limited data available for Indonesia on the serological prevalence and risk factors of HBV infection in HCWs, and describes the need to mount a nationwide policy for the prevention, control, and management of hepatitis B in HCWs.

## SEROPREVALENCE OF HBV INFECTION AMONG HCWs IN INDONESIA

**Fig. 1:**
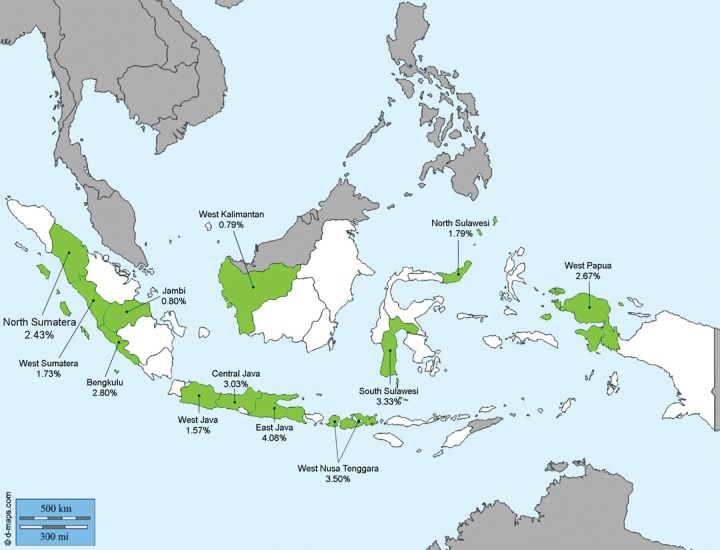
Prevalence of HBsAg among HCWs in 12 provinces in Indonesia 2015 (courtesy of the Subdirectorate of Hepatitis and Gastrointestinal infection, Directorate of Direct Communicable Diseases)

Efforts to collect data for HBV infection among health care personnel in Indonesia have been made by the Subdirectorate of Hepatitis and Gastrointestinal infection, Directorate of Direct Communicable Diseases, Ministry of Health, Republic of Indonesia, which examined the prevalence of HBsAg among 60,000 HCWs from 12 provinces in Indonesia.^13^ The average HBsAg prevalence was 2.56%, ranging from 2.5 to 21.6% (Fig. 1). This figure was lower than the national prevalence of HBsAg in the general population.

**Graph 1:**
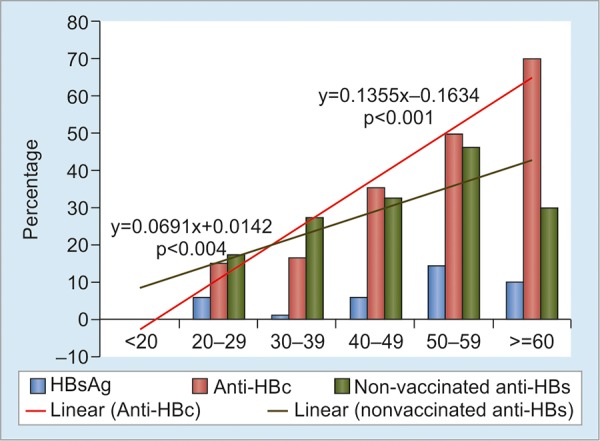
The prevalence of HBsAg, anti-HBc, and anti-HBs among 464 HCWs according to age groups (<20, 20-29.9, 30-39.9, 40-49.9, 50-59.9, and >60 years). Linear-by-linear association test showed significant increasing trends of anti-HBc (p < 0.001) and nonvaccinated anti-HBs (HBsAg-, anti-HBc+, anti-HBs+) (p = 0.004) by age indicating accumulated exposure to HBV infection by increasing age. No significant difference was found in the prevalence of vaccinated anti-HBs among the three groups

**Graph 2:**
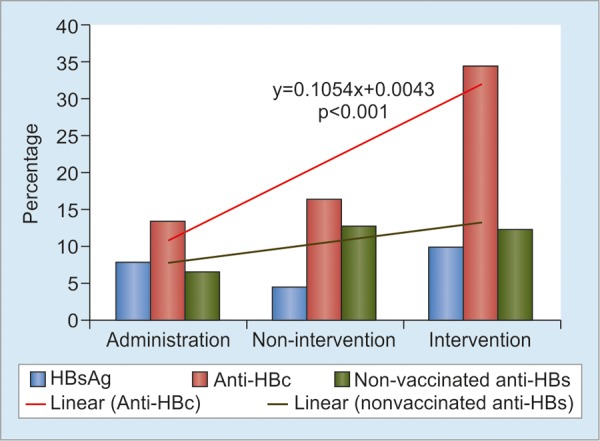
The prevalence of HBsAg, anti-HBc, and anti-HBs among 644 HCWs according to types of work: Administration (19.1%), nonintervention (63.6%), and intervention (17.3%). Linear-by-linear association test showed significant increasing trends of anti-HBc (p < 0.001). No trend was observed for nonvaccinated anti-HBs

**Graph 3:**
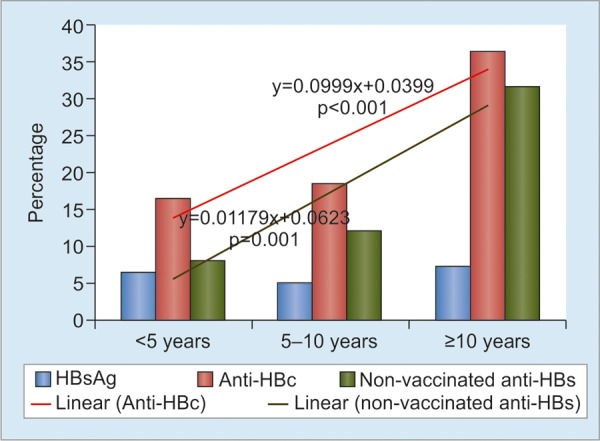
The prevalence of HBsAg, anti-HBc, and anti-HBs among 644 HCWs according to length of service period (<5, 5-10, and >10 years) in Indonesia. Linear-by-linear association test showed significant increasing trends of anti-HBc (p < 0.001) and nonvaccinated anti-HBs (p = 0.010). No significant difference was found in the prevalence of vaccinated anti-HBs among the three groups

A specific study was done in 2017 on 644 HCWs (male/female 134/510; median age 28, 16-71 years) from four areas in South Sulawesi Province (Luwu Timur, Palopo, Makassar, and Bantaeng) and one hospital (Gading Pluit) in Jakarta.^14^ They were categorized into three types of work: Administration (administrative and technical service staff), nonintervention (doctors, nurses, and other personnel who were not exposed to materials contaminated with patient’s blood or body fluid in their routine work), and intervention (HCWs with exposure-prone procedures, such as surgeons, dentist, gynecologist, and laboratory personnel). The prevalence of HBsAg, anti-HBc, and anti-HBs was 4.7, 18.5, and 36.7% respectively, while 57.3% were negative for all seromarkers, indicating susceptibility to this infection. In the study, HBV infection rate was measured by examining the evidence of exposure (anti-HBc) and the marker of resolved infection (HBsAg-, anti-HBc+, anti-HBs+) as an indicator for repeated natural boosting. These markers significantly increased with age, suggesting accumulated exposure to HBV infection by increasing age (Graph 1). Significant rising trend of anti-HBc positivity was observed across three types of work (Graph 2): administration, nonintervention, and intervention (HCWs with exposure-prone procedures, such as surgeons, dentist, gynecologist, and laboratory staff). The exposure and resolved-infection rates also markedly increased with the length of service period, suggesting increasing occupational risk of HCWs to HBV infection (Graph 3).

## RISK FACTORS FOR ACQUISITION OF HBV AMONG HCWs

A number of variables are associated with acquisition of HBV infection among HCWs. A higher rate of HBV exposure has been reported in older HCWs than in younger ones, as found in the above and other studies.^10,14,15^ One explanation could be that there is a more constant risk of exposure during the lifetime that increases the hepatitis B prevalence with age. Another important factor is the type of work that shows higher infection rates in HCWs with exposure-prone profession, such as laboratory staff, dentists, surgeons, and gynecologists.^9,10,14,15^ In addition, long employment in health care services increases the risk of acquiring HBV infection.^9-11,14,15^

As consistently reported, the most common mode of exposure to HBV infection is through sharp injury, which occurs mostly in the intervention group. In the United States, during 1997 to 1998, more than 385,000 needle-stick injuries occurred annually.^16^ In the United Kingdom, 37% of nurses had sustained a needle-stick injury at some stage during their career.^17^ In Indonesia, a study in 2007 found 48% of 377 HCWs experienced at least one sharp injury, with rates of 64% among obstetrics-gynecology staff and 30% among the interns,^12^ while the recent 2017 study confirmed needle-stick injury as a high-risk factor (OR 1.707; p = 0.029) to get infected.^14^ Even though HCWs have repeated a given procedure so many times, one slip can cause injury with potentially serious consequences; an unexpected or sudden movement by the patient or a transient lack of concentration can result injury.^18^

## PROTECTING AND MANAGEMENT OF HBV INFECTION IN HCWs

### Standard Precautions

The World Health Organization (WHO)^19^ and the Joint United Nations Programme on HIV/AIDS have recommended the application of standard precautions to protect HCWs from blood-borne pathogens including human immunodeficiency virus (HIV) and hepatitis.

Standard precautions combine the major features of Universal Precautions and body Substance Isolation and are based on the principle that all blood, body fluids, secretions, excretions (except sweat), nonintact skin, and mucous membranes may contain transmissible infectious agents. Standard precautions include a group of infection-prevention practices that apply to all patients, regardless of suspected or confirmed infection status, in any setting in which health care is delivered. These include: Hand hygiene; use of gloves, gown, mask, eye protection or face shield, depending on the anticipated exposure; and safe injection practices. Also, equipment or items in the patient environment likely to have been contaminated with infectious body fluids must be handled in a manner that prevent transmission of infectious agents (e.g., wear gloves for direct contact, contain heavily soiled equipment, properly clean and disinfect or sterilize reusable equipment before use on another patient).

Standard precautions are also intended to protect patients by ensuring that health care personnel do not carry infectious agents to patients on their hands or via equipment used during patient care.

### Education, Training, and Proper Work Schedule

Effective and educational programs targeting at HCWs need to be implemented.^6,18^ Lack of training in infection prevention, safety guidelines as well as long working hours significantly elevate the odds of exposure to risky conditions.^18,20^ It was reported that working for more than 48 hours per week significantly increased occupational exposures to HIV/acquired immunodeficiency syndrome risky conditions in one year compared with those who had worked less or equal to 48 hours per week.^21^ Working excessive hours can result in stress and emotional and physical exhaustion, which are likely to increase the chance of human error and contribute to a tendency toward risky behaviors and poor adherence to the precautions.^18^

### Vaccination

Considering the high exposure rates to HBV infection, HCWs highly need vaccination in order to be protected. This is in line with the recent WHO updated position paper on hepatitis B vaccine—July 2017, which recommends that HCWs and other groups with occupational exposure should be the targets for vaccination. It is emphasized that hepatitis B vaccination safeguards health workers when administered early, ideally before occupational exposure. A catch-up strategy targeted at HCWs who are still susceptible to HBV infection is also needed.^3,5,10^ Although routine postvaccination testing is not recommended, persons at risk of occupational exposure to HBV infection should be considered for postvaccination testing to ensure that they have achieved a protective anti-HBs level (≥10 mIU/mL).^22^ It is also recommended that HCWs should be periodically monitored for their immune status (e.g., every five years) and booster doses should be given to those who have become susceptible (anti-HBs level <10 IU/mL).^5^

### Postexposure Prophylaxis

Postexposure prophylaxis for HBV requires the evaluation of several factors, such as the HBsAg status of the source, and the vaccination and HBV immunity status of the exposed HCW. After any exposure, the unvaccinated HCW should receive a single dose of HBV immunoglobulin, preferably within 24 hours of exposure, followed by the HBV vaccination series.^4,16^

### Management of HBV-infected HCWs

Being infected with HBV should not preclude entry into a health care profession. However, public safety and the right of the HCWs with HBV to practice without loss of the right of confidentiality about their health status should be at the right balance.^4^ Those who are HBsAg positive and undertake exposure-prone procedures, such as surgeons, gynecologists, nurses, phlebotomists, and dentists, should be considered for antiviral therapy to reduce direct transmission to patients.^3^ In conformity with the 2013 antiretroviral recommendations,^23^ they should receive a potent antiviral agent with a high barrier to resistance (i.e., entecavir or tenofovir) to reduce levels of HBV deoxyribonucleic acid, ideally to undetectable or at least to 2000 IU/mL, before resuming exposure-prone procedures.^3,4^

### Guidelines and Policy

Guidelines and recommendations for the prevention, control, and management of HBV infection in HCWs have been issued in several countries^9^; however, this is not widely implemented in most low- and-middle-income countries despite hepatitis B endemicity.^3^ Recognizing this particular problem, the Global Health Sector Strategy on Viral Hepatitis 2016 to 2020 that was adopted by the World Health Assembly in May 2016 has stated occupational health as the core intervention and priority actions for countries to end viral hepatitis from being a major public health threat.^24^ More recently, the WHO Regional Office for South-East Asia in July 2017 has included routine hepatitis B vaccination among HCWs as a strategic direction in the Regional Action Plan for Viral Hepatitis 2016 to 2021.^8^ Drawing upon these two WHO strategic documents, it is targeted that all Member States have started implementation of routine hepatitis B vaccination among high-risk groups including HCWs by 2020.^8,24^

Given that most countries including Indonesia have implemented hepatitis B vaccination among infants, subgroups of adults at high risk, such as HCWs should be considered as a matter of policy.^3,10,22^ For example, vaccination against HBV could be made mandatory for preclinical medical and nursing students. In addition, education on infection control and other strategies need to be strengthened.^6^ Establishment of surveillance systems for registering, reporting, and management of occupational exposures in health institutions are required in countries with hepatitis B endemicity.^3,4,18^

## CONCLUSION

The HCWs are at high occupation risk to HBV infection, which is associated with the age, type of work, and length of service period of HCWs, particularly in the professions that routinely perform exposure-prone procedures. In all studies reported, sharp injuries contributed the highest risk of getting HBV infected. There is an urgent need to safeguard the HCWs with hepatitis B vaccination, which further provides greater protection to patients from infection through exposure to infected health workers. Infection control needs to be strengthened and continuing education has to be imparted to all HCWs at various health care setups. The establishment of a national policy and a roadmap for effective and efficient intervention is required for the prevention, diagnosis, postexposure management, and treatment of HBV infection in this special population.
